# Single-nucleus RNA sequencing reveals dynamic changes in the microenvironment of visceral adipose tissue and metabolic characteristics after cold exposure

**DOI:** 10.3389/fendo.2025.1562431

**Published:** 2025-03-24

**Authors:** Ting Yi, Shuai Wu, Yusha Yang, Xi Li, Shuran Yang, Yongqiang Zhang, Li Zhang, Yuyu Hu, Guanyu Zhang, Jun Li, Danfeng Yang

**Affiliations:** ^1^ School of Public Health, The Key Laboratory of Environmental Pollution Monitoring and Disease Control, Ministry of Education, Guizhou Medical University, Guiyang, China; ^2^ Academy of Military Medical Sciences, Academy of Military Sciences, Tianjin, China; ^3^ School of Chinese Materia Medica, Tianjin University of Traditional Chinese Medicine, Tianjin, China

**Keywords:** cold exposure, visceral adipose tissue, snRNA-seq, metabolic homeostasis, cellular heterogeneity

## Abstract

**Introduction:**

Visceral adipose tissue (VAT) plays a crucial role in regulating systemic metabolic balance. Excess accumulation of VAT is closely associated with various metabolic disorders, a process that involves the coordinated actions of multiple cell types within the tissue. Cold exposure, as a potential intervention, has been proposed to improve metabolic dysfunction. However, the heterogeneity of VAT and its comprehensive metabolic characteristics under cold exposure remain unclear.

**Methods:**

We collected epididymal white adipose tissue (eWAT) of C57BL/6J mice after cold exposure at three different time points for single-nucleus RNA sequencing (snRNA-seq) analysis.

**Results:**

We successfully identified ten major cell types in eWAT, enabling understanding of the dynamic changes in the eWAT microenvironment and its metabolic features during cold exposure. This study revealed that cold exposure for 1 day reduced cellular metabolic activity and intercellular communication in eWAT including receptor-ligand-based cell communication and metabolite-mediated interactions. However, after 14 days of cold acclimation, the metabolic activity of adipocytes was significantly enhanced, and intercellular metabolic communication was restored. Additionally, prolonged cold exposure promoted the formation of a distinct adipocyte subpopulation that may be associated with UCP1-independent thermogenesis. These changes may be a new homeostatic state established by VAT to adapt to the cold environment. The study also identified the importance of adipocytes, adipose stem and progenitor cells, myeloid cells, and endothelial cells in the process of cold adaptation.

**Discussion:**

This research provides valuable insights into the cellular heterogeneity, adipocyte remodeling, and metabolic reprogramming in eWAT after cold exposure. It highlights the critical role of transcriptional dynamics in eWAT during cold exposure and provides new perspectives on the prevention and treatment of metabolic diseases.

## 1 Introduction

Nowadays adipose tissue has become widely recognized as an active metabolic organ having both endocrine and immunological functions, as well as a high degree of plasticity, rather than as an inert organ for energy storage as was once thought (1). It secretes a range of biologically active peptides and adipokines that communicate with the surrounding tissues and organs in a paracrine, autocrine, or endocrine manner, which is essential for maintaining the metabolic homeostasis of the organism (2).

Based on developmental origin, morphology and functional differences, adipose tissue can be categorized into three types: brown adipose tissue (BAT), white adipose tissue (WAT) and beige adipose tissue (Beige) (3). WAT can be classified into two categories based on anatomical location: subcutaneous white adipose tissue (sWAT) and visceral adipose tissue (VAT), each having distinct pathophysiological functions. inguinal white adipose tissue (iWAT) is a specialized subtype of sWAT. It possesses a unique ability to undergo thermogenic conversion. VAT is located in the abdominal cavity around organs such as the liver, kidneys, pancreas and gonads, and excessive accumulation of VAT is a high-risk factor for obesity co-morbidities such as obesity, diabetes mellitus, cardiovascular disease and atherosclerosis (4).

Currently, metabolic syndrome due to obesity has reached pandemic levels globally and is widely recognized as a major public health issue worldwide (5). Therefore, there is an urgent need to formulate novel preventative approaches, control tactics, and better lifestyles to reduce fat accumulation and alleviate the economic and societal burden of obesity-related ailments. The involvement of VAT is involved in regulating metabolic homeostasis within the body, involving various cell types such as adipocytes, adipose stem cells and progenitors (ASPCs), immune cells, endothelial cells, and mesothelial cells (6). Moreover, appropriate cold exposure can ameliorate metabolic disorders by inducing changes in VAT metabolism. Tissue remodeling is one of the early responses of VAT adaptation to cold environments, indicating that VAT is capable of responding to low temperatures (7). According to the findings, cold exposure activates the body’s adaptive thermal production mechanisms to promote metabolic health (8). Uncoupling protein 1(UCP1) is the key molecular target that promotes adaptive thermogenesis. Cold exposure activates UCP1 in brown and beige adipocytes, releasing the energy used to synthesize ATP in the form of heat energy (9). Although UCP1 has been recognized as the primary contributor to thermogenesis in adipose tissue, several seminal studies in recent years have demonstrated the existence of UCP1-independent thermogenesis mechanisms, including Ca^2+^ cycling, creatine cycling, and TAG/FA cycling in adipocytes (10–13). The core principle of UCP1-independent thermogenesis is futile cycling, in which an ATP-consuming reaction occurs simultaneously with a counteracting energy reaction, leading to heat dissipation (11). This process is catalyzed by a series of enzymes that drive substrate cycles, converting chemical energy into heat through ATP hydrolysis. Notably, this pathway has been shown to be involved the regulation of systemic energy homeostasis (13). These important findings provide new perspectives on anti-obesity treatment. Nevertheless, the characteristics of changes in the VAT microenvironment, its metabolic heterogeneity, and the interactions and regulatory mechanisms between different cell populations after cold exposure are poorly understood, which largely limits the possibilities of treating metabolic diseases in the future.

In recent years, single-cell RNA sequencing (scRNA-seq) has significantly advanced our understanding of tissue heterogeneity (14, 15). However, due to the size limitations of microfluidic systems, which are unable to process cells with diameters exceeding 60 micrometers, scRNA-seq encounters challenges when characterizing lipid-laden adipocytes. Consequently, previous studies have primarily focused on exploring the heterogeneity of adipose stem cells and immune cells (16–18). Recently, snRNA-seq has been applied to analyze the adipocytes (19). The technique measures RNA molecules in the nucleus of individual cells, circumventing the unsuitability of scRNA-seq in large-sized adipocytes. This contributes to understanding the heterogeneity within adipocytes and their interactions with stromal vascular cells. Today, snRNA-seq technology is mainly used for analysis in BAT and inguinal white adipose tissue (iWAT), as well as eWAT in obese mouse models. For example, Sun et al. (20) identified a subset of Cyp2e1^+^ adipocytes with thermogenic regulatory functions in BAT. Liu et al. (21) highlighted intracellular changes during the browning process of iWAT under chronic cold exposure (6°C). Another study used snRNA-seq to further explore the heterogeneity of adipocytes in iWAT after aging and cold exposure over different time periods (22). Sárvári et al (23). analyzed the effects of obesity on adipocyte changes in eWAT subgroups. However, snRNA-seq datasets for VAT after cold exposure are relatively scarce, limiting our comprehensive understanding of how cold exposure regulates metabolic processes in VAT.

To characterize the heterogeneity of cells and their metabolic changes in mouse VAT under different cold exposure times. We performed snRNA-seq analysis on eWAT from mice subjected to varying durations of cold exposure (Day0, Day1, and Day14). Our data revealed the cellular landscape of eWAT under different cold exposure periods. To examine the metabolic remodeling in eWAT, we quantified its metabolic activity using scFEA analysis. Adipocyte metabolic activity was higher in groups exposed to cold for Day14 than in groups exposed to cold for Day0 and Day1. Furthermore, We further revealed the heterogeneity of adipocytes and their functional and biological characteristics in eWAT after cold exposure.

Remarkably, Day14 exhibited an increase in a type of cell population that undergoes *de novo* lipogenesis (DNL) and contributes to UCP1-independent thermogenesis, compared to the other experimental groups. Our study also emphasized the functional differences and regulatory mechanisms of transcription factors across adipocyte subpopulations. Finally, we systematically explored cellular interactions and metabolite exchanges between adipocyte subpopulations and other cell types in the tissue. In summary, these results reveal the role of different cell types in eWAT, particularly adipocytes, in regulating metabolic processes, including fatty acid metabolism, energy expenditure, and glucose metabolism, during cold exposure of varying durations. Our findings will contribute to a more comprehensive understanding of the function of VAT and provide insights for subsequent studies exploring the mechanisms of metabolism-related diseases as well as targeted defense strategies.

## 2 Materials and methods

### 2.1 Animals and samples

The male C57BL/6J mice, aged 8 weeks, were obtained from Beijing Vital River Laboratory Animal Technology Co. Ltd. (Beijing, China). They were maintained in a controlled environment with a 12-hour light/dark cycle and humidity levels ranging from 45% to 60%. Mice were given water and a standard laboratory chow diet (Rat & Mouse Growth and Reproduction Formula Feed, Keao Xieli Feed Co.,Ltd, Tanjing, China) ad libtum unless otherwise specified. After 1 week of acclimatization at room temperature (22-24°C), the mice were randomly divided into groups based on body weight (n=12 per group), including the Day0 group, Day1 group, and Day14 group. Before cold exposure, all groups were placed at thermoneutrality (30°C) for 1 week, then at 4°C for 0, 1, or 14 days, respectively. At the end of the experiment, mice were euthanized and eWAT was collected. All procedures related to animal care and use were approved by the Committee on the Ethics of Animal Experiments of the Academy of Military Medical Science (AMMS-04-2022-027).

### 2.2 Equations single-nucleus RNA sample preparation, library construction, and sequencing

eWAT from male C57BL/6J mice subjected to different durations of cold exposure was isolated. Each group consisted of three replicates. A 100 mg frozen adipose sample was placed on dry ice, and all sample processing was performed on ice until nuclear isolation. Nuclei were extracted using the BioYoo Nuclear Isolation Kit (SHBIO, #52009-10, Shanghai, China). Counted using a Luna-FL automated cell counter (Logos Biosystems, Gyeonggi-do, Korea). According to the manufacturer’s protocol, single-nucleus gene expression libraries were constructed using the Chromium Next GEM Single Cell 3′ Reagent Kits v3.1 (10× Genomics, Pleasanton, CA, USA), and libraries were sequenced using an Illumina NovaSeq 6000 sequencing system.

### 2.3 Single-nucleus RNA data processing

Data were processed using the Cell Ranger toolkit (10× Genomics, v.7.1.0) with default parameters. Illumina sequencing output FASTQs files were aligned to the mouse reference genome GRCm38 version of mouse genome. A genetic barcode matrix was generated for each sample by calculating UMI and filtering out non-cell-related barcodes, and the resulting gene expression matrix was subsequently used for downstream analysis.

The gene expression matrices from nine mouse samples were then imported into the Seurat (v5.0.1) package for quality control and downstream analysis of snRNA-seq data. Unless otherwise specified, all functions were run using default parameters. Cells with fewer than 200 or more than 5000 genes, fewer than 10,000 UMI counts, more than 15% mitochondrial gene expression, or more than 2% ribosomal gene expression were excluded. Doublets were removed using DoubletFinder (v2.0.4). Data normalization, standardization, dimensionality reduction, clustering, and batch effect removal were performed using NormalizeData, ScaleData, FindVariableFeatures, RunPCA, and RunHarmony. During initial clustering, as observed in previous adipose single-cell datasets, contaminating epididymal cells (*Adam28*, *Adam7*, *Spef2*), sperm-derived nuclei (*Dnah12*, *Hydin*, *Abcc9*), and epithelial cells (*Dcdc2a*) were removed (23, 24). FindClusters (resolution = 0.3) was used for cell clustering. Uniform Manifold Approximation and Projection (UMAP) was used to visualize clustering results. The FindAllMarkers function was used to identify marker genes for each cell cluster. For each cluster, differential expression genes (DEGs) were defined using the Wilcoxon rank-sum test. Marker genes were selected based on *p*-value adjusted < 0.05 and |log_2_FC| > 0.25. For the re-decimation clustering of adipocyte populations, after further removing low-quality cell clusters, we determined the optimal clustering resolution through a systematic approach. First, principal component analysis (PCA) was performed on adipocytes, identifying 13 principal components (PCs). Next, Clustree visualization was applied to examine the clustering tree across resolutions from 0 to 1, alongside UMAP projection to further assess clustering stability. Finally, we identified cluster-specific marker genes and integrated these findings to determine the optimal clustering resolution of 0.2. KEGG and GO enrichment analyses were performed on DEGs using the ClusterProfiler package. Visualize the results using the ggplot2 package. Cell types were identified using the top 50 genes ranked by mean fold change in expression alongside established marker genes from the literature.

### 2.4 Single-cell flux estimation and cell metabolite prediction

The metabolic flux rate of mice cells was estimated using the graph neural network algorithm based on Python (3.8) single cell flux estimation analysis (scFEA) (25).

### 2.5 Trajectory analysis

RNA velocity analysis was conducted by using scVelo (version 0.3.2). In particular, to count spliced and unspliced reads for each sample, the 10 × velocyto pipeline was run in the filtered cell ranger-generated BAM files, while for single-nucleus RNA velocity inference, the dynamical model of scVelo was applied (26).

### 2.6 Cell-cell interaction analysis

To explore the ligand-receptor interactions between cells in the eWAT microenvironment with different temporal cold exposures, we used the CellChat package (27) for further analysis.

### 2.7 Cell-cell metabolic communication

Metabolite-mediated cellular communication events between adipocyte subpopulations and other cell populations were further inferred by using Python (3.8) MEBOCOST (28) analyses.

### 2.8 Network analysis for transcriptional regulators

The pySCENIC algorithm was used to construct gene regulatory networks for assessing the activity of transcription factor (TF) regulators in adipocyte subpopulations (29). The connectivity specificity index (CSI) parameter was employed for module analysis, as reported in previous studies.

### 2.9 Statistical analysis

All statistical analyses were conducted using R (version 4.3.2). The Wilcoxon rank-sum test was employed to identify differentially expressed genes between different samples and cells, followed by Bonferroni *post-hoc* analysis. *p*-value adjusted < 0.05 were considered statistically significant.

## 3 Results

### 3.1 snRNA-seq reveals the cell atlas of eWAT in mice after cold exposure

**Figure 1:**
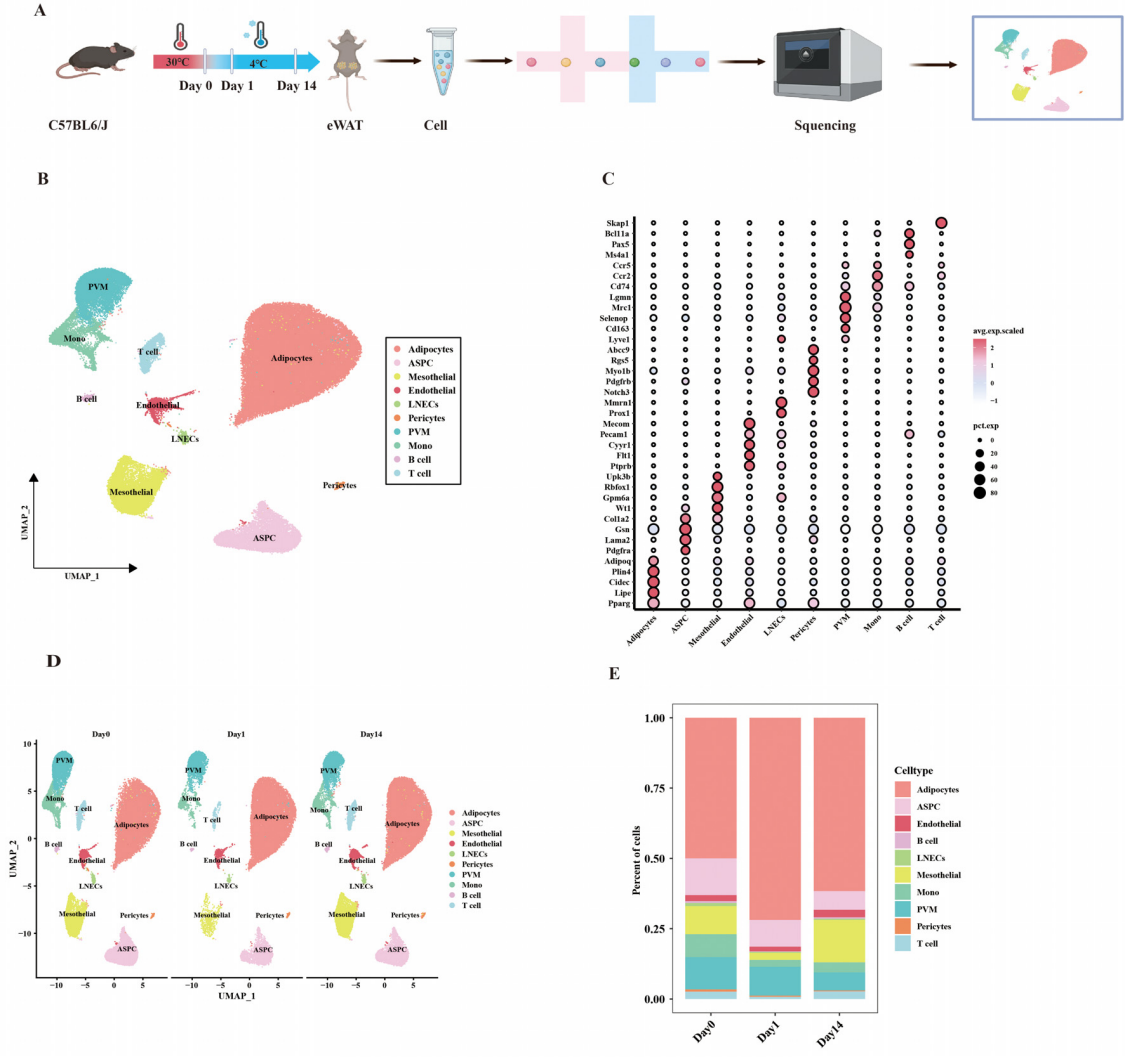
UMAP clustering analysis of single-cell data from mouse eWAT under different durations of cold exposure. **(A)** Schematic overview of the snRNA-seq experimental workflow. **(B)** UMAP clustering plot showing the partitioning of cell types in eWAT, colored by cell type. **(C)** Dot plot showing the average expression levels of classic marker genes associated with cell types. **(D)** UMAP plot displaying the distribution of cell subpopulations at different time points. **(E)** Bar plot showing the proportion of each cell type at different time points.

To investigate the contribution of VAT to cold adaptation, we performed snRNA-seq on eWAT from mice at three-time points: Day0 (thermoneutrality, 30°C, 7 days), Day1 (cold exposure, 4°C, 1 day), and Day14 (cold exposure, 4°C, 14 days) (**Figure 1A**). Following quality control, a total of 88022 cells were acquired, comprising 30384 cells from Day 0, 19785 cells from Day 1, and 37853 cells from Day 14. Unsupervised clustering and annotation based on known lineage markers revealed 10 major cell types. These include adipocytes (*Adipoq*, *Plin4*, *Cidec*, *Lipe*, *Pparg*), adipose stem cell and progenitor cell (ASPC; *Col1a2*, *Gsn*, *Lama2*, *Pdgfra*), mesothelial cell (*Wt1*, *Gpm6a*, *Rbfox1*, *Ubk3b*), endothelial cell (*Ptprb*, *Flt1*, *Cyyr1*, *Pecam1*, *Mecom*), lymphatic endothelial cells (LNECs; *Prox1*, *Mmrn1*), pericytes (*Noth3*, *Pdgfrb*, *Myo1b*, *Rgs5*, *Abcc9*), perivascular macrophage (PVM; *Lyve1*, *Cd163*, *Selenop*, *Mrc1*, *Lgmn*), monocyte (Mono; *Cd74*, *Ccr2*, *Ccr5*), B cell (*Ms4a1*, *Pax5*, *Bcl11a*), and T cell (*Skap1*) (**Figures 1B, C**). Cold exposure at different times did not lead to the disappearance of any cell types, but the percentage of each cell type varied slightly. For example, adipocytes showed an increase after day 1 of cold exposure and presented a decrease after 14 days of cold exposure. (**Figures 1D, E**). The above results suggest that adipocytes undergo remodeling after cold exposure at different times. This remodeling may reflect an adaptive response of visceral adipose tissue to cold stimuli.

### 3.2 Cellular metabolic heterogeneity in eWAT after cold exposure

**Figure 2:**
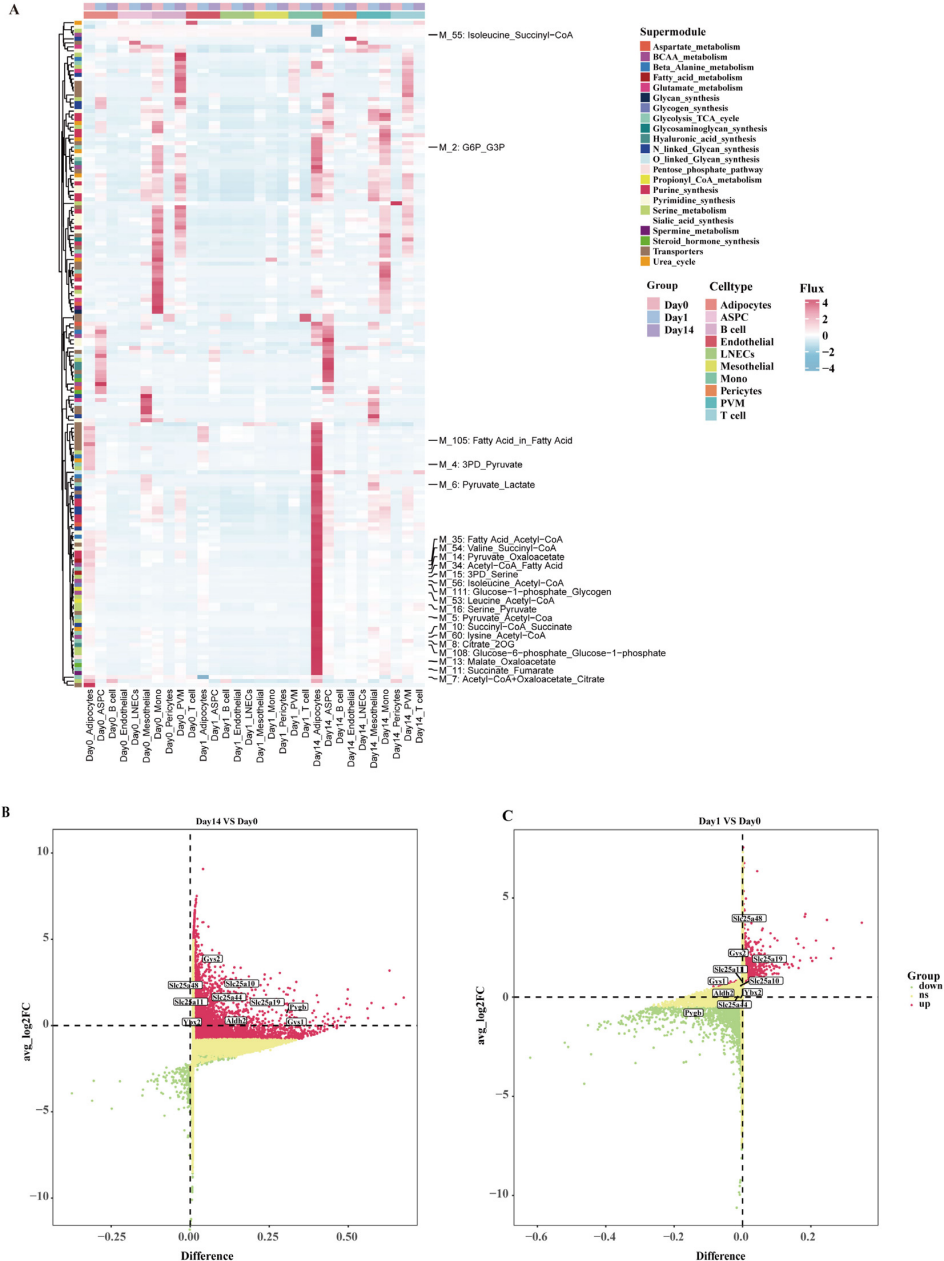
The metabolism landscape of eWAT under different durations of cold exposure. **(A)** Heatmap showing the average metabolic of the flux profiles in different cell types. **(B, C)** Volcano plot shows differentially expressed genes in adipocytes at various time points. Differentially expressed metabolic enzymes and transporters are highlighted in the figure.

Cellular metabolic interactions and heterogeneity are considered crucial for maintaining organismal homeostasis. To comprehensively reveal the key metabolic features in eWAT during cold exposure, we utilized an integrated method called scFEA to systematic evaluation of the effects of cold exposure at different time points on 168 metabolic modules and 70 metabolites at the single-cell level (**Supplementary Table S1**). The study result indicates that 1 day of cold exposure leads to a decrease in cellular metabolic activity within eWAT. Notably, M_7 (acetyl coenzyme A+ oxaloacetate_citrate), which is associated with the initial phase of the tricarboxylic acid (TCA) cycle showed a significant reduction in activity within adipocytes. In contrast, after 14 days of cold exposure, the metabolic activity of eWAT cells was enhanced to varying degrees, with the most significant increase observed in the adipocytes. This included glycolysis + TCA cycle, fatty acid metabolism, BCAA catabolism, and transporter proteins (**Figure 2A**). The involved metabolic modules were primarily related to glucose metabolism, including M_2 (G6P_G3P), M_4 (3PD_Pyruvate), M_5 (Pyruvate_Acetyl-CoA), M_6 (Pyruvate_Lactate), M_8 (Citrate_2OG), M_10 (Succi-yl-CoA_Succinate), M_11 (Succinate_Fumarate), M_13 (Malate_Oxaloacetate), M_14 (Pyruvate_Oxaloacetate), M_71 (Glucose_in_Glucose), M_111 (Glucose-1-phosphate_Glycogen), M_108 (Glucose-6-phosphate_Glucose1-pho-sphate). These results suggest that adipocytes may adapt to cold environments by altering the reprogramming of glucose metabolism. Of note, the activity of modules related to BCAA catabolism was increased in adipocytes after 14 days of cold exposure, including M_53 (Leucine_Acetyl-CoA), M_54 (Valine_Succinyl-CoA), M_56 (Isoleucine_Acetyl-CoA), M_60 (Lysine_Acetyl-CoA). Previous studies have shown that the accumulation of BCAA can lead to the development of various metabolic diseases (30). We further investigated the differential expression of enzymes and transporter proteins in adipocytes under different cold exposure conditions. The Day14 group significantly upregulated key metabolic enzymes involved in the catabolism of BCAAs, in particular the mitochondrial solute carriers *Slc25a10*, *Slc25a11*, *Slc25a19*, and *Slc25a44 (* 31). Concurrently, enzymes related to glycogen metabolism in the M111 module, including *Gsy1*, *Gys2*, and *Pygb*, also exhibited upregulation (**Figures 2B, C**). In conclusion, our study demonstrates that after 14 days of cold exposure, adipocytes achieve metabolic homeostasis through enhanced glucose metabolism and BCAA catabolic activity, highlighting a critical mechanism for eWAT adaptation to the cold environment.

### 3.3 Cold exposure induces adipocyte remodeling

**Figure 3:**
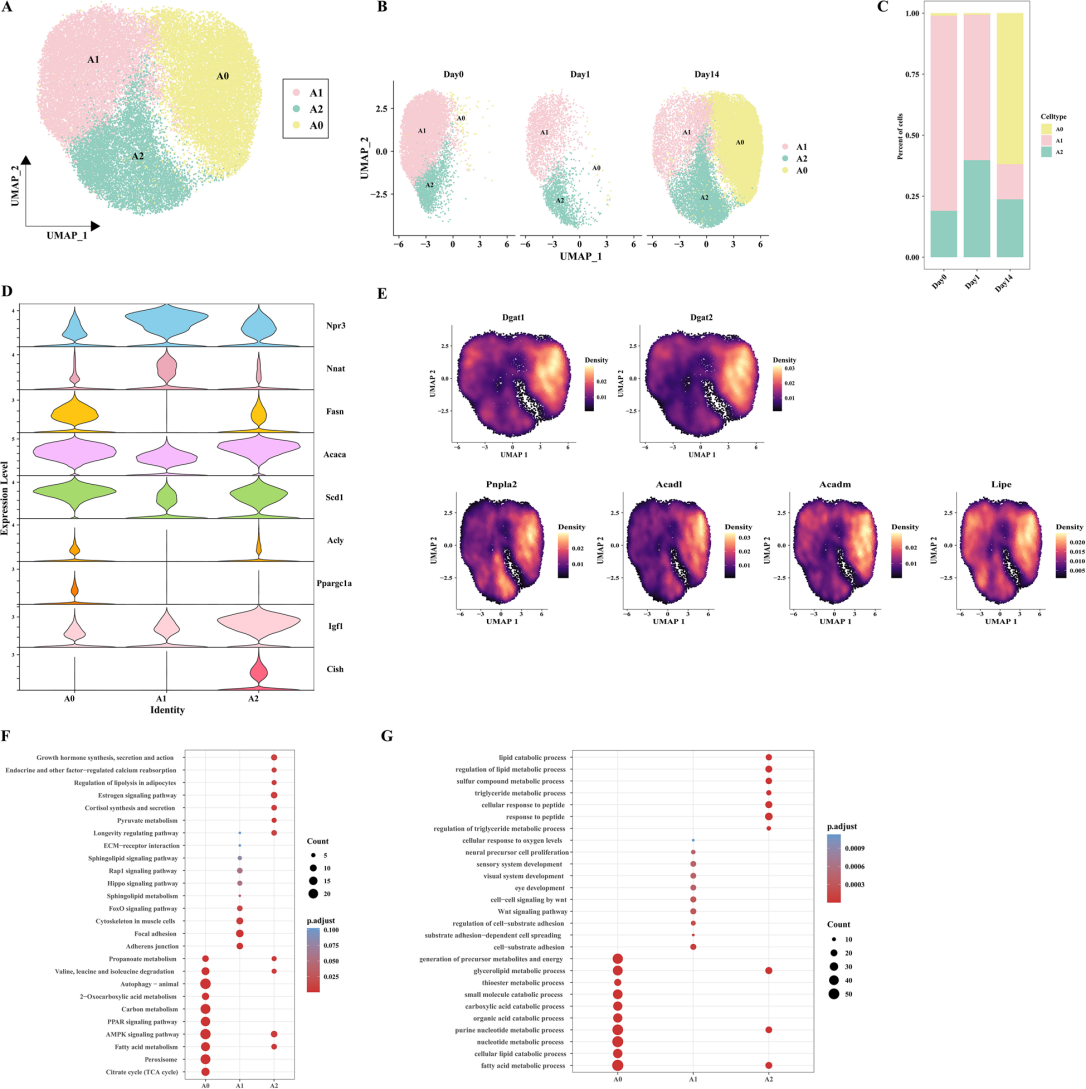
Characteristics of adipocyte subpopulations in mice at different time points. **(A)** UMAP clustering of adipocyte clusters. **(B)** UMAP plot showing the distribution of adipocyte subpopulations at different conditions. **(C)** Bar plot displaying the proportion of adipocyte subpopulations at different time points. **(D)** Violin plot depicting the expression patterns of specific genes in adipocyte populations. **(E)** Cloud plot showing the density distribution of genes associated with TAG synthesis (Dgat1, Dgat2), lipolysis (Pnpla2, Lipe) and fatty acid oxidation (Acadl, Acadm) on UMAP. **(F)** Bubble plot displaying the KEGG enrichment analysis of the three adipocyte subpopulations. **(G)** Bubble plot displaying the GO enrichment analysis of the three adipocyte subpopulations.

Given the high abundance of adipocytes in the three groups and the observed influence of cold exposure on adipocyte metabolic activity, we further investigated the phenotypic characterization of adipocyte subpopulations. After quality control, we reclustered the adipocytes (n=33897) and identified three distinct adipocyte subpopulations: A0, A1, and A2 (**Figure 3A**). We observed that the A0 subpopulation was predominantly found in the Day14 group. The proportion of subpopulation A1 decreased in the Day14 group compared to the Day0 group, and the proportion of subpopulation A2 increased in the Day1 group (**Figures 3B, C**). To further investigate potential differentiation relationships among the three adipocyte subpopulations, RNA velocity analysis was conducted to capture transcriptional transient dynamics within the cells. The results indicated that no differentiation relationship was observed among the three adipocyte subpopulations (**Supplementary Figure 1**). All three subpopulations highly expressed classical adipocyte markers (*Plin4*, *Plin1*, *Pparg*, *Adrb3*) (**Supplementary Figure 2A**). We noticed that *Npr3* and *Nnat* were enriched in the A1 subpopulation but lacked *Acly*, similar to the lipid-scavenging adipocytes (LSA) identified by Sárvári et al. (23) and the ‘Npr3-high’ adipocytes described by Holman et al (22). Both the A2 and A0 subpopulations expressed genes associated with DNL, such as *Fasn*, *Acaca*, *Scd1*, and *Acly*. These observations are reminiscent of the A5 adipocytes discovered in cold-exposed BAT by Patrick et al (32). This result shows that lipid biosynthesis in eWAT is mediated by the A0 and A2 subpopulations. In addition, the feature that distinguishes the A0 from A2 is that A0 subpopulation expresses the *Ppargc1a* (encoding PGC-1α), which is associated with thermogenesis. It is indicated that a potential thermogenic pathway exists in the A0 subpopulation. In contrast, the A2 subpopulation showed high expression of *Igf1* and *Cish* (**Figure 3D**). This result showed that A2 may be associated with cellular growth processes. Similar Cish-high cell subpopulations have also been described in the report of Sun et al (20). The A0 and A2 subpopulations may signify two functional states of the same adipocyte subpopulation under varying temperature circumstances. However, UCP1^+^ adipocytes were not observed, which may be attributed to the tissue’s insensitivity to cold-induced sympathetic nervous system activation (33). Recent studies have identified a UCP1-independent thermogenic adipocyte subpopulation in iWAT under cold exposure, characterized by high expression of *Atp5k* and involvement in creatine and lipid cycling metabolism (34). UCP1-independent thermogenic mechanisms contribute to systemic energy and glucose homeostasis. A recent study utilizing Ucp1 KO mouse models demonstrated that lipid futile cycling plays a crucial role in adaptive thermogenesis and total energy expenditure (13). Furthermore, chronic β3-adrenergic agonist treatment has been shown to increase the basal metabolic rate in Ucp1 KO mice, particularly within eWAT (35). Notably, cold-induced adipose browning is generally absent in the VAT of lean, healthy mice, and VAT rarely exhibits UCP1-dependent adaptive thermogenesis (33). The discovery of UCP1-independent thermogenic pathways presents promising opportunities for combating obesity and improving metabolic health. We sought to explore whether a UCP1-independent thermogenesis pathway exists in eWAT adipocytes. We performed heatmap enrichment analysis of genes associated with UCP1-independent thermogenesis in adipocyte subpopulations (**Supplementary Figure 2B**). Of note, we observed that genes related to Ca²^+^ futile cycling and TAG/FA futile cycling (*Atp2a2*, *Dgat1*, and *Dgat2*) were enriched in the A0 subpopulation. However, no significant expression of creatine futile cycling-related genes (*Slc6a8*, *Ckm*) was detected. Given the high expression of DNL-related genes in the A0 subpopulation, we further hypothesize that the A0 subpopulation may be involved in TAG/FA futile cycling-mediated thermogenesis. To test this hypothesis, we examined the distribution of genes related to lipogenesis (*Dgat1*, *Dgat2*), fatty acid oxidation (*Acadm*, *Acadl*), and lipolysis (*Pnpla2*, encoding *ATGL*; and *Lipe*, encoding *HSL*) in adipocyte subpopulations. The results showed a significant increase in the expression of these genes in the A0 subpopulation (**Figure 3E**). These findings suggest the existence of a UCP1-independent thermogenic pathway mediated by TAG/FA futile cycling in eWAT. These findings suggest that there may be a UCP1-independent thermogenic pathway mediated by the TAG/FA futile cycling in eWAT.

To determine the biological characteristics of each adipocyte subpopulations, KEGG and GO enrichment analyses were performed on their differentially expressed genes. The A0 subpopulation primarily performs metabolic functions, participating in pathways such as BCAA catabolism (valine, leucine, and isoleucine degradation), carbon metabolism, PPAR signaling pathway, AMPK signaling pathway, fatty acid metabolism, and citric acid cycle (TCA cycle). The A1 subpopulation is closely associated with cellular structures and signaling such as intercellular adhesion junctions, the FoxO signaling pathway, and cytoskeleton in muscle cells. The A2 subpopulation is primarily involved in the growth hormone synthesis, secretion and action, endocrine and other factor-regulated calcium, regulation of lipolysis in adipocytes, estrogen signaling pathway, cortisol synthesis and secretion, longevity regulating pathway, suggesting that A2 may plays a pivotal role in preserving endocrine homeostasis in eWAT (**Figures 3F, G**).

### 3.4 The cell-cell communication of eWAT during cold exposure

**Figure 4:**
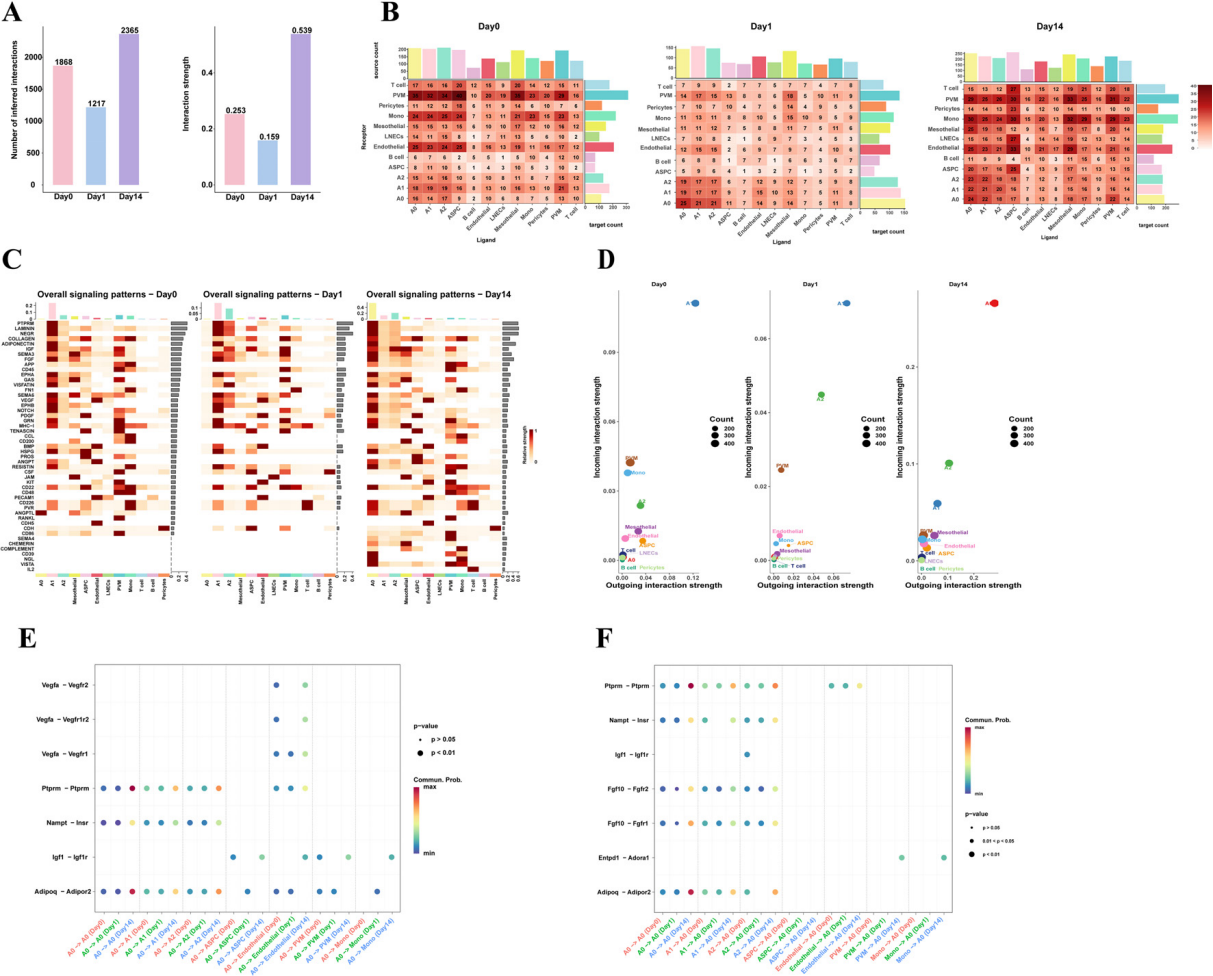
Cell communication networks in eWAT under different timepoints of cold exposure. **(A)** The number and strength of cell-cell communications in mouse eWAT at different time points after cold exposure. **(B)** Heatmap showing the total number of potential ligand-receptor pairs between different cell types at each timepoint, obtained using CellChat. Bar graphs represent the sum of the columns or rows. **(C)** Heatmap showing the overall signaling pathway activity at different time points. The y-axis represents signaling pathways, and the x-axis represents cell subpopulations. The color intensity in the heatmap indicates the strength of communication, with darker colors representing stronger signaling. The bars on the top and right side represent the cumulative strength of the signals along the respective axes. **(D)** Scatter plot visualizing the major signal receivers and senders in different cell populations under cold exposure at different time points. **(E, F)** Dot plot displays 7 pairs of ligand-receptors selected across different time points between A0 adipocytes and adipocyte subpopulations, ASPC, endothelial, and myeloid cells (PVM, Mono). (**(E)**, A0 as the receiver; **(F)** A0 as the sender). The color of the dots represents the communication probability.

Cellular interactions are crucial for maintaining normal tissue function. To explore the mechanisms of cell-to-cell regulation under different cold exposure conditions, we used CellChat to identify complex signaling communication patterns between cells in eWAT under three cold exposure conditions, constructing a cell-cell interaction network. We first compared the number and strength of intercellular interactions during cold exposure and observed a trend of initial decrease followed by an increase in eWAT. Specifically, 1868, 1217, and 2365 intercellular interactions were identified on Day 0, Day 1, and Day 14, with corresponding interaction intensities of 0.253, 0.159, and 0.539, respectively (**Figure 4A**).

Interestingly, further analysis of the intercellular interaction networks under different experimental conditions revealed that cell communication between adipocyte subpopulations increased with prolonged cold exposure. In contrast, communication between non-adipocyte cell types decreased after 1 day of cold exposure but increased after 14 days, with PVM, monocytes, endothelial cells, and ASPC participating in the majority of interactions (**Figure 4B**). These results suggest that adipocytes, myeloid cells, endothelial cells, and ASPC are key cell types in eWAT’s adaptation to cold environments. To identify the main functional cell types at different cold exposure time points, we compared the overall signaling patterns, outgoing, and incoming interaction strengths of each cell group, we found that in the Day0 group, the A1 subpopulation was the main sender and receiver of signals. In the Day1 group, the A1 and A2 subpopulations play a dominant role in both signaling transmission and reception, while in the Day14 group, the A0 subpopulation was the primary senders and receivers (**Figures 4C, D**). This indicates that different adipocyte subpopulations perform distinct functions and roles under different cold exposure conditions. The A0 subpopulation may be the key cell type in eWAT response to long-term cold exposure.

Subsequently, we performed ligand-receptor pair analysis for adipocyte subpopulations, endothelial cell, ASPC, PVM and Mono. When A0 acted as the signaling sender (ligand), A0 adipocytes interacted with other adipocyte subpopulations via autocrine/paracrine signaling through PTPRM-(Ptprm-Ptprm), VISFATIN- (Nampt-Insr), and ADIPONECTIN- (Adipoq-Adipor2). The signaling intensity in the Day14 group was significantly stronger than that in the Day0 and Day1 groups. Additionally, A0 can also interact with myeloid cells (PVM/Mono) through IGF- (Igf1-Igf1r). Previous studies have shown that IGF1 derived from adipose tissue macrophages (ATM) in the perigonadal fat is critical for maintaining adipose tissue mass during cold exposure (36). A0 adipocytes mediate most vascular activities through interactions with endothelial cells via VEGF signaling pathways, including Vegfa-Vegfr2, Vegfa-Vegfr1r2, and Vegfa-Vegfr1. It has been established that the plasticity of adipose tissue requires continuous vascular growth and remodeling (37). From the perspective of the signal receiver, we observed that the A2 subpopulation can interact with the A0 subpopulation via IGF- (Igf1-Igf1r). Myeloid cells can interact via CD39 (Entpd1-Adora1), a pathway that was only present in the Day14 group (**Figures 4E, F**). Overall, adipocytes in eWAT are actively responding to the cold environment and preparing for remodeling.

### 3.5 Metabolite exchange in cells within eWAT after cold exposure

**Figure 5:**
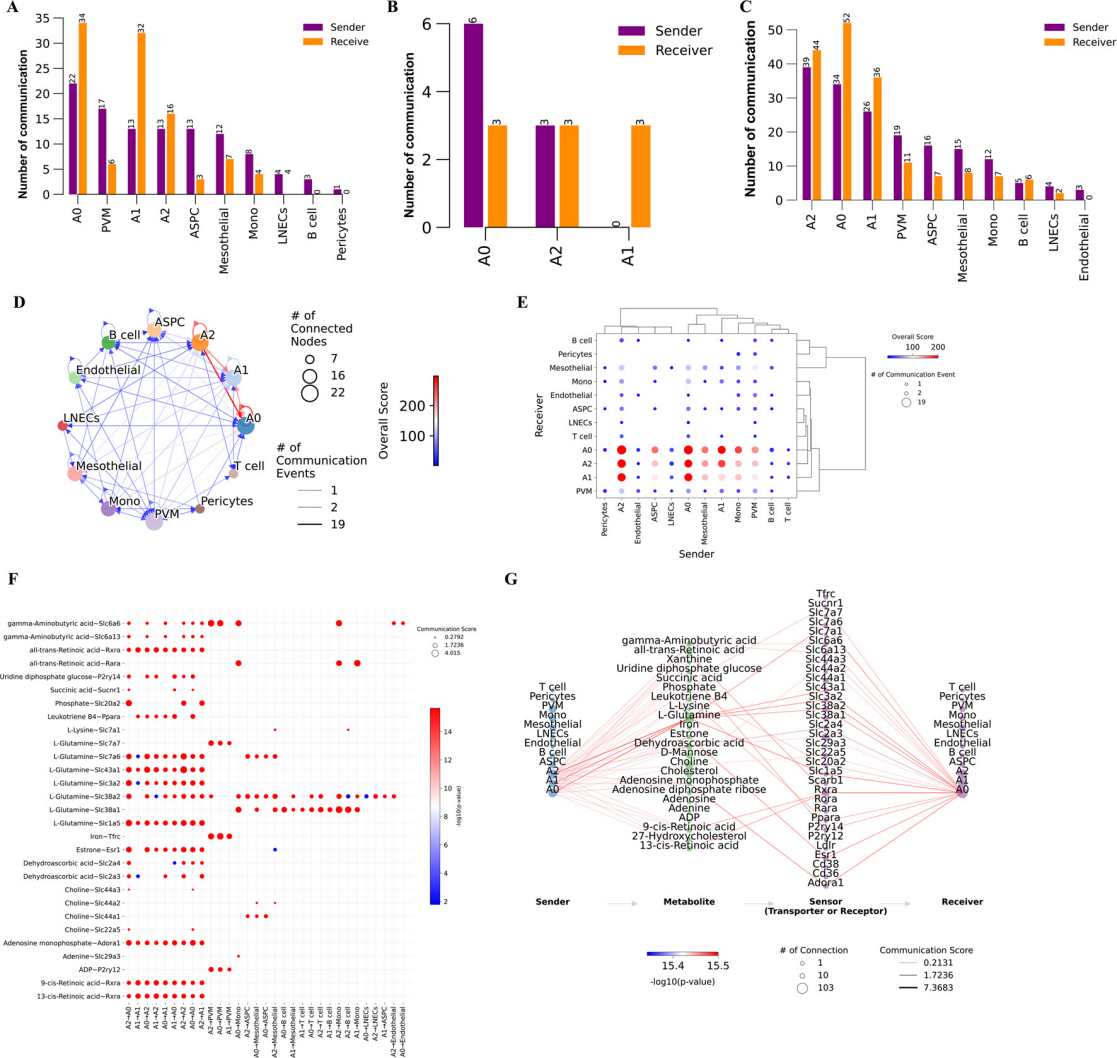
Metabolic communication networks between eWAT cells under different timepoints of cold exposure. **(A–C)** Bar charts illustrating the quantity of communications detected between each type of cell as a signal sender and receiver under Day 0 **(A)**, Day 1 **(B)**, and Day 14 **(C)** conditions. **(D)** Network diagram of metabolite-sensor communication between eWAT cells at Day 14. Each point represents a cell type. **(E)** Dot plot illustrating communication between each pair of cell types. **(F)** Dot plot showing metabolite-sensor communication sent from adipocyte subpopulations to adipocytes and other cell types. **(G)** Flow diagram of the metabolite-sensor communication between adipocyte subpopulations.

VAT is a crucial endocrine organ that plays a central role in the development of various metabolic diseases, with adipogenesis being regulated by metabolites (38–40). Additionally, the above scFEA analysis revealed that adipocytes exhibit significantly higher metabolic activity after 14 days of cold exposure. Based on these observations, we hypothesize that distinct cell types in eWAT after cold exposure communicate via metabolites, thereby modulating the metabolic homeostasis of the tissue microenvironment. To verify the above hypothesis, we employed the MEBOCOST algorithm to infer the metabolic interaction patterns and calculate metabolite abundance for each single-cell subpopulation. Our analysis showed that in the Day 0 group, we found that adipocyte subpopulations, PVM, Mono, ASPC, mesothelial cell, pericytes, LNECs, and B cell communicate through metabolites. Adipocytes exhibited strong metabolic communication under all three conditions. Notably, in the Day 1 group, only metabolic communication between adipocyte subpopulations was observed. By the Day14 group, metabolic communication in myeloid cells, ASPC, and endothelial cells was restored, with endothelial cell metabolic communication displaying unique features in the Day14 group (**Figures 5A–C**). These findings align with the results from CellChat analysis, indicating that these cell types play important roles in the body’s adaptive response to cold exposure. Together, these results suggest that intercellular metabolite-sensor communication in mouse eWAT is modulated by environmental temperature. Given the potential thermogenic function of the A0 subpopulation and its significance in eWAT adaptation to prolonged cold exposure, this study focused on the metabolic communication in the Day 14 group, which includes a greater diversity of adipocyte subpopulations. This approach allows us to explore the metabolic changes within eWAT during cold adaptation, and shedding light on the critical role of cold exposure in improving metabolic homeostasis. We observed that autocrine signaling was more frequent than paracrine signaling in adipocyte subpopulations, and the A0 subpopulation exhibited the most frequent communication. (**Figures 5D, E**). By plotting a metabolite-sensor communication dot plot between cells, we found that adipocyte subpopulations mainly communicated through glutamine and retinoic acid. The A2 subpopulation primarily regulates phosphate metabolism through the secretion of Slc20a2, a process that occurs exclusively among adipocyte subpopulations. Phosphate is involved in various physiological processes, including energy production and storage, intercellular signal transduction, and cell growth. Sufficient phosphate levels regulate lipid homeostasis by modulating fatty acid biosynthesis and oxidation. Our results suggest that prolonged cold exposure promotes the A2 subpopulation to regulate lipid homeostasis in eWAT through phosphate metabolic pathways (41). PVM could absorb iron from adipocyte subpopulations via Tfrc (**Figure 5F**). Reducing iron levels in adipocytes has been shown to decrease body weight, reduce fat mass, improve glucose tolerance, and enhance insulin sensitivity in obese mouse models (42). When only the A0 subpopulation was considered as the receptor, it is noteworthy that A0 more prominently absorbed glutamate from the A2 subpopulation via Slc1a5 (**Figure 5G**). The above results suggest that eWAT improves metabolic health and promotes cold acclimatization of the body by regulating metabolite exchanges between cells.

### 3.6 Differences in transcriptional regulation of adipocyte subpopulations after cold exposure

**Figure 6:**
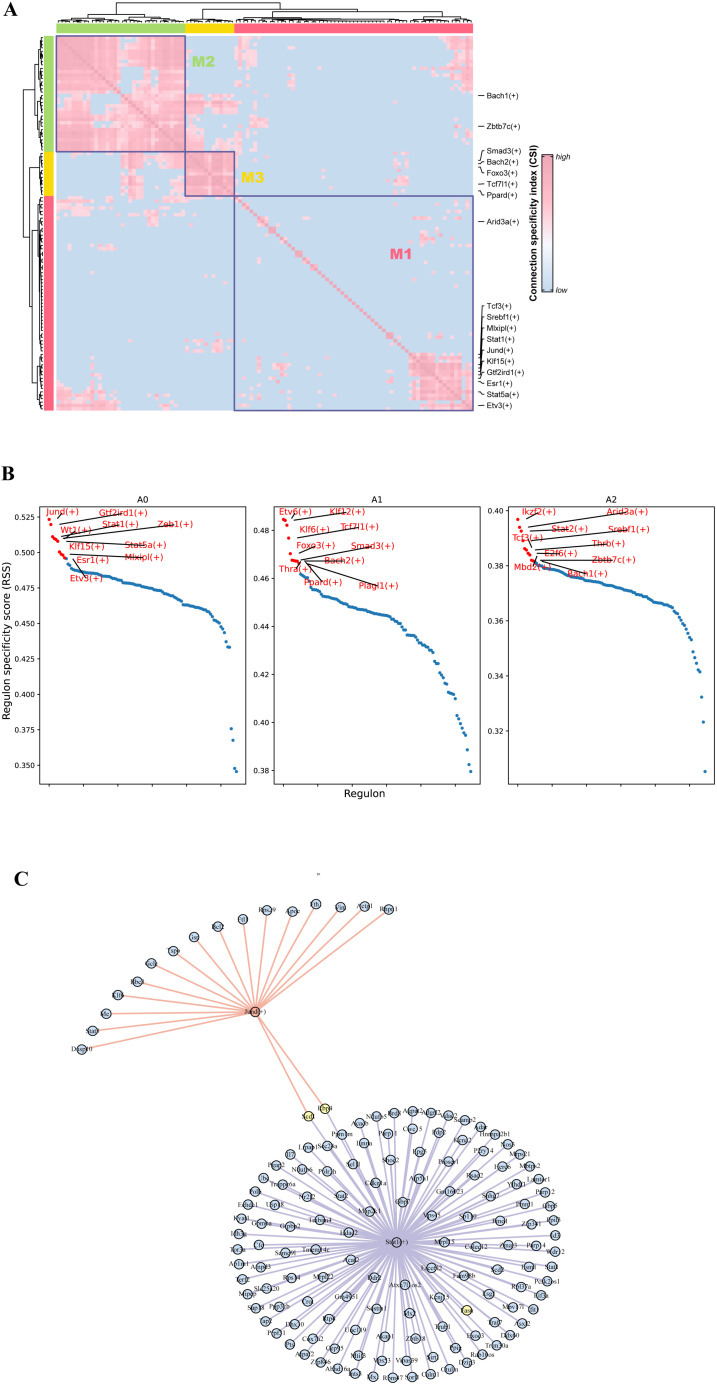
Transcription factor regulatory features of different adipocyte subpopulations. **(A)** Regulator modules are identified based on the CSI. **(B)** Specificity scores of regulators in different adipocyte subpopulations. **(C)** Regulatory network of transcription factors Jund and Stat1 and their target genes.

All of the above results underscore the critical role of adipocyte subpopulations in facilitating cold acclimation and highlight that different adipocyte subpopulations perform different functions. Furthermore, cellular states are maintained by gene regulatory networks composed of transcription factors and their target genes. To explore the transcriptional regulatory mechanisms underlying adipocyte state changes after cold exposure at different time points, we performed pySCENIC analysis on different adipocyte subpopulations. A total of 110 important regulators, including 7067 target genes, were identified (**Supplementary Table S2**). Transcription factors typically work together to regulate gene expression levels. To systematically characterize the combinatorial patterns, we compared the range similarity of regulon activity score (RAS) scores for each regulator pair using the CSI. RAS is the Regulon activity score for each cell calculated by the AUCell method. It measures the enrichment of a regulon in a particular cell. A higher RAS indicates that the TF and its target genes are more active in that cell. The results showed that the 110 regulators were divided into three major modules (M1, M2, M3). Transcription factors involved in adipogenesis were mainly enriched in the M1 module, while transcription factors of the A1 subpopulation appeared in the M3 module (**Figure 6A**). By analyzing the average activity score ranking of the three modules in each cell subpopulation, we found that the A0 subpopulation was mainly distributed in the M1 module (**Supplementary Figure 3**). The M1 module included the largest number of regulators, totaling 63, including Mlxipl, Srebf1, Stat1, Stat5a, Jund, and others, which have been previously reported to play important roles in adipogenesis (43–46). This suggests that the M1 module and its downstream target genes collectively participate in regulating adipocyte generation. Regulon specificity score (RSS) is a measure of the specificity of a regulon in a particular cell subpopulation; the higher the RSS, the more specific the Regulon is in that subpopulation. The top 10 specific regulators for each adipocyte subpopulation were identified based on RSS, including Jund, Sta5a, Stat1, Mlxipl, and Gtf2ird1, which are specific transcription factors for the A0 subpopulation. Klf12, Tcf7l1, Etv6, and Foxo3 are specific transcription factors for the A1 subpopulation. Arid3a, Stat2, and Srebf1 are specific transcription factors for the A2 subpopulation. Further interaction network analysis revealed that Fasn is regulated by Stat1, while Jund and Stat1 jointly regulate the transcription of Scd1 (**Figure 6C**). Interestingly, Mlxipl was found to regulate Slc25a44, which is involved in BCAA clearance (**Supplementary Figure 4**). Combining the scFEA analysis results, the enhanced BCAA catabolic activity after 14 days of cold exposure is likely driven primarily by the A0 subpopulation.

## 4 Discussion

Cold exposure promotes lipid turnover in adipocytes and induces DNL in adipose tissue (47). Prior research has demonstrated that concurrent fatty acid oxidation and DNL in brown adipocytes can produce a thermogenic pathway characterized by a TAG/FA futile cycle (13). DNL in VAT is crucial for maintaining glucose and lipid metabolic equilibrium (48). Therefore, understanding the heterogeneity of visceral adipocytes and their potential metabolic changes during cold exposure is important for developing therapeutic strategies for metabolic diseases.

This study utilized snRNA-seq to delineate the transcriptome of mouse eWAT at an unprecedented single-nucleus resolution after 0, 1, and 14 days of cold exposure, resulting in a comprehensive cellular atlas of eWAT. The resulting dataset is also available as a public repository. Ten cell types were identified in eWAT after cold exposure including adipocytes, ASPC, endothelial cell, LNECs, PVM, Mono, B cell, and T cell. We utilized scFEA and MEBOCOST to infer cellular metabolic activity and metabolite-mediated intercellular communication in VAT at the single-cell level. Notably, the integration of these two approaches to investigate cold exposure-induced metabolic changes in VAT at the single-cell level has not been previously reported. This enables a comprehensive understanding of the cell communication patterns between adipocytes and other cell types in eWAT after cold exposure. The identified cell types and their gene expression changes during cold exposure provide valuable insights for future therapeutic strategies targeting metabolic diseases.

Growing research indicates that the dynamic remodeling and metabolic adaptation of adipose tissue are essential for responding to changing cold environments (49, 50). Consistently, our metabolic flux analysis revealed that eWAT adapts to cold exposure by modulating intracellular metabolic transitions in adipocytes. At day 1 cold exposure, a marked reduction in cellular metabolic activity was observed in eWAT. Notably, the activity of M_7 (acetyl-CoA + oxaloacetate_citrate), which is associated with the initial phase of the tricarboxylic acid (TCA) cycle, was significantly reduced in adipocytes, highlighting the critical role of the TCA cycle during the early stage of cold exposure. In contrast, after 14 days of cold exposure, adipocytes exhibited enhanced metabolic activity, particularly in glucose metabolism, fatty acid metabolism, and BCAA catabolism. Differential enzyme and protein expression analysis revealed that cold exposure enhances BCAA clearance from the circulatory system by upregulating Slc25a44, promoting BCAA uptake and oxidation, thereby supporting metabolic health. This mechanism has been validated in BAT during acute cold exposure (51). We argue that the response rate of adipose tissue to external environmental changes may differ depending on its anatomical location, and the regulation of metabolic homeostasis in VAT requires prolonged cold exposure. For the first time, we mapped the metabolic landscape of eWAT under cold exposure at the single-cell level.

Previous studies have performed snRNA-seq analysis on mouse eWAT under specific conditions such as aging, cold exposure and obesity to reveal the heterogeneity and gene expression profile of adipocytes (22, 23, 32). In contrast, the heterogeneity of eWAT adipocytes and their panoramic metabolic features under different durations of cold exposure remain unclear, and the collaborative or unique contributions of adipocytes and other cell types to metabolic homeostasis have not been explored. This study identified three adipocyte subpopulations present during cold exposure: A0, A1, and A2. The A1 subpopulation is distinguished by elevated expression of Npr3 and Nnat, both of which inhibit thermogenesis in adipocytes and are associated with obesity. The number of this subpopulation decreases after cold exposure, suggesting a potential role of cold exposure in ameliorating central obesity. Furthermore, A0 represents a distinct adipocyte subpopulation, present solely in the Day 14 group, that mediates UCP1-independent thermogenesis via the TAG/FA futile cycle. In our study, we observed an enrichment of genes related to TAG/FA metabolism in the A0 subpopulation, whereas creatine futile cycling-related genes (*Slc6a8*, *Ckm*) did not exhibit significant expression levels. However, we observed that the A0 adipocyte subpopulation expressed the key gene Atp2a2, which is involved in Ca²^+^ futile cycling. Although studies have suggested that TAG/FA futile cycling plays a predominant role in thermogenesis in UCP1KO mice exposed to cold (13). However, the regulatory process of thermogenesis involves multiple pathways and is not governed by a single mechanism. Currently, the coordinated regulation among different futile cycling-based thermogenic pathways remains unclear, making it an important avenue for future investigation. A deeper understanding of these mechanisms may provide potential strategies for improving metabolic health. In contrast, the A2 subpopulation predominantly serves endocrine functions. Furthermore, pathway enrichment analysis of the A2 subpopulation suggested its potential involvement in longevity regulation. Cold exposure has been reported to be associated with rejuvenation effects and improved metabolism (52). It promotes longevity by reducing chronic inflammation, enhancing antioxidant capacity, increasing energy expenditure, and altering metabolic pathways (53). Recent studies have further revealed that adipocytes, as potent endocrine cells, can exert profound effects on neighboring tissues and organs through the secretion of bioactive peptides and extracellular vesicles, thereby regulating aging and lifespan (54). Similarly, Zaczek et al. highlighted the critical role of visceral fat in longevity and metabolic health maintenance (55). Additionally, iron metabolism dysregulation has been implicated in aging progression and lifespan regulation (56). Our findings indicated that perivascular macrophages (PVMs) are capable of absorbing iron from adipocytes, suggesting that the A2 adipocyte subpopulation may play a potential role in longevity regulation. However, we have not yet experimentally validated the changes or functions of A2 adipocytes in aging models. Further studies are needed to elucidate their precise role in aging and longevity regulation.

Transcription factor analysis suggests that these changes in cellular states may be due to differences in the regulators of each adipocyte subpopulation. RSS analysis identified Mlxipl and Gtf2ird1 as specific transcription factors of the A0 adipocyte subpopulation. ChREBP (gene name: Mlxipl) is a key transcription factor regulating *de novo* lipogenesis (DNL) and plays a crucial role in maintaining glucose homeostasis. Under conditions of nutrient excess, ChREBP-regulated DNL in adipocytes can exert beneficial effects on metabolic homeostasis (57). Our target gene network analysis suggested that Slc25a44 may be a downstream target of Mlxipl. Previous studies have shown that Slc25a44 in adipose tissue enhances metabolic health by regulating BCAA catabolism (51). Additionally, the DNA-binding transcription factor Gtf2ird1 forms a complex with Prdm16 and Ehmt1, which effectively inhibits adipose tissue fibrosis and improves systemic glucose homeostasis (58). Notably, this glucose homeostasis regulation occurs independently of UCP1-mediated thermogenesis. These findings provide key insights into how adipocytes respond to external stimuli through different metabolic mechanisms, thereby regulating metabolic homeostasis.

Cell-cell communication plays a crucial role in regulating metabolic homeostasis. We constructed a ligand-receptor-based cell communication network between different cell types in mouse eWAT. Additionally, we employed MEBOCOST to fill the gap in metabolite communication studies based on snRNA-seq. Predicting cell-cell metabolite-sensor interactions after cold exposure at different time points to reveal new dimensions in metabolic mechanism research. We observed an interesting phenomenon: intercellular communication under both modes was attenuated on Day 1, except in the adipocyte subpopulation. Cold exposure on Day 14 restored intercellular communication. Zhang et al (59). observed a similar phenomenon in perirenal adipose tissue (PRAT), where acute cold exposure at 4°C for 3 days reduced intercellular communication in PRAT, while enhancing communication in iWAT and iBAT. The reduction in intercellular communication on day 1 may represent a protective response, as the organism has not yet fully adapted to the cold environment. At this point, the organism may reduce intercellular communication to prevent a rapid decline in core body temperature. This phenomenon may be specific to visceral adipose tissue (60, 61). The research we have performed showed that the recovery of cell communication may requires a 14-day cold acclimation period. However, it remains unclear whether intercellular communication could be restored in a shorter time frame. Additionally, in the Day 14 group, interactions within the ADIPONECTIN signaling pathway between adipocyte subpopulations were significantly enhanced. Studies have confirmed that adiponectin is a critical regulator of thermogenesis and is essential for maintaining body temperature during cold exposure. This may reflect the body’s attempt at this specific time point to regulate inflammation, oxidative stress, and insulin resistance through enhanced adiponectin signaling, thereby promoting a healthier state (62). This suggests that during prolonged cold exposure, the body may enhance adiponectin signaling to maintain body temperature, lipid metabolic homeostasis, and insulin sensitivity. This is essential for sustaining metabolic equilibrium and preventing metabolic diseases. In terms of metabolic communication, after day 14 cold exposure, adipocyte subpopulations primarily communicate through glutamine and retinoic acid. Research indicates that glutamine is a key carbon source for DNL production and serves as an important energy substrate for BAT thermogenesis after cold exposure (63, 64). Retinoic acid, a metabolite of vitamin A, is the key active compound involved in physiological processes. Previous studies have demonstrated that Vitamin A transport plays a critical role in adipose tissue browning and thermogenesis after cold exposure (65). Moreover, 9-cis-retinoic acid has been shown to promote UCP1 expression in brown adipocytes through RXR activation (66). These findings suggest that glutamine and retinoic acid may emerge as promising candidates for inducing thermogenesis in visceral adipocytes in the future. Interestingly, after day 14 cold exposure, PVMs can uptake iron from adipocyte subpopulations through Tfrc. Iron metabolism adaptation has been reported to be a key determinant of thermogenic function in adipocytes. Adequate iron accumulation in adipocytes enhances thermogenesis, while iron uptake by resident macrophages, acting as a “ferrostat”, limits local iron availability, thereby suppressing adipocyte browning (67–69). In contradiction to this, iron overload in adipocytes has been linked to an increased risk of diabetes (70). Other investigations suggest that reducing iron levels in adipocytes promotes healthier WAT (42, 71). In conclusion, PVM plays a critical role in finely regulating intracellular iron levels in adipocytes, which is essential for maintaining systemic metabolic health. Future studies are needed to explore the specific mechanisms by which PVM regulate iron content in adipocytes after cold exposure, and how this impacts adipocyte browning and glucose metabolism homeostasis, to fully understand its role in metabolic health. Furthermore, previous studies have demonstrated that specific secreted proteins, such as Peptidase M20 Domain Containing 1 (PM20D1), catalyze the formation of N-acyl amino acids from fatty acids and amino acids. These metabolites function as mitochondrial membrane-associated uncouplers, facilitating UCP1-independent thermogenesis (72). Recent research further revealed that the ligand glucose-dependent insulinotropic polypeptide (GIP) activates its receptor GIP receptor (GIPR), inducing SERCA-mediated futile calcium cycling in adipocytes, thereby contributing to thermogenesis (73). Notably, glucagon-like peptide-1 (GLP-1) receptor agonists have been shown to exert significant effects in antidiabetic and anti-obesity treatments (74). These findings collectively suggest that UCP1-independent thermogenesis can be achieved through specific proteins or metabolites, offering promising clinical applications, which represents an important area for further investigation in future research. Overall, this study provides valuable insights into the metabolic adaptations of eWAT and metabolite-mediated intercellular communication. The findings described herein may offer new perspectives for clinical interventions in metabolic disorders and the identification of novel therapeutic targets.

This study has several limitations that should be acknowledged. Firstly, compared to scRNA-seq, snRNA-seq detects fewer immune cells, which may limit our ability to fully understand the role of immune cells in regulating eWAT metabolic homeostasis. Future studies should incorporate scRNA-seq data analysis to comprehensively capture and analyze the diversity and function of immune cells, potentially providing important insights into the regulation of metabolic homeostasis. Secondly,

Notably, our study is based on transcriptomic data inference, and the identification of a potential UCP1-independent thermogenic pathway in the A0 subpopulation requires further validation. Future studies should employ flow cytometry or genetic labeling techniques to isolate A0 cells and experimentally verify their functional and metabolic activities, particularly in relation to UCP1-independent thermogenesis. Although our study identifies the A0 subpopulation as a potential target for metabolic disease therapy, further research using clinical samples is required due to differences between human and animal models. Notably, there is no consistency in the heterogeneity of mouse and human adipocytes (24). In addition, existing literature suggests low reproducibility of human adipocyte subpopulations across studies (75), which reflects the inherent biological variability of adipocytes. In future studies, the presence of A0 or A2 like adipocytes in human adipose tissue needs to be further explored by combining clinical samples or publicly available datasets. Furthermore, the intercellular communication analysis cannot control for potential confounding factors, thus further functional validation is needed to elucidate the underlying mechanisms.

It should be noted that environmental temperature changes can influence gene expression by altering DNA methylation, histone modifications, and other epigenetic mechanisms, thereby enhancing insulin sensitivity (76). Taylor et al (77) conducted reduced representation bisulfite sequencing (RRBS) on BAT samples collected from mice exposed to thermoneutral conditions, room temperature, and cold exposure for two weeks. Their findings revealed that chronic cold stress affects DNA methylation in BAT, potentially modulating transcription, splicing, and histone modifications to facilitate adaptive responses. The methylation status of CpG islands may play an important role in the dynamics of gene expression in adipocytes, especially during the transition between short-term and long-term adaptation to cold exposure. Our study primarily focuses on preliminarily investigating the gene expression characteristics of different adipocyte subpopulations during cold exposure. As an early exploration of VAT adaptations at the single-cell level, we have not yet performed a systematic CpG island analysis across different adipocyte subpopulations. However, we recognize that this represents a promising direction for further research. In future studies, we aim to employ ATAC-seq, RRBS, or whole-genome bisulfite sequencing to investigate DNA methylation dynamics across different adipocyte subpopulations, providing deeper insights into the epigenetic mechanisms underlying cold adaptation in VAT.

In summary, this study provides a single-nucleus resolution cell atlas of mouse eWAT after cold exposure at different time points and elucidates the metabolic characteristics of adipocytes. We highlight the potential cell interactions and metabolic exchange between adipocytes and other cell types during DNL. Our work contributes to understanding the metabolic changes in eWAT adipocytes under cold exposure. Furthermore, this work may provide potential avenues for targeted interventions in the treatment of metabolic diseases.

## Data Availability

The datasets presented in this study can be found in online repositories. The names of the repository/repositories and accession number(s) can be found below: https://www.ncbi.nlm.nih.gov/, PRJNA1199939.
