# Supplementary material for: Single-nucleus RNA sequencing reveals dynamic changes in the microenvironment of visceral adipose tissue and metabolic characteristics after cold exposure
Source: Front Endocrinol (Lausanne). 2025 Mar 24;16:1562431. doi: 10.3389/fendo.2025.1562431 (PMC11973077; doi:10.3389/fendo.2025.1562431)
Supplement: Supplementary file 2 [file DataSheet2.docx]

Supplementary Material

# Supplementary Figures and Tables

## Supplementary Figures


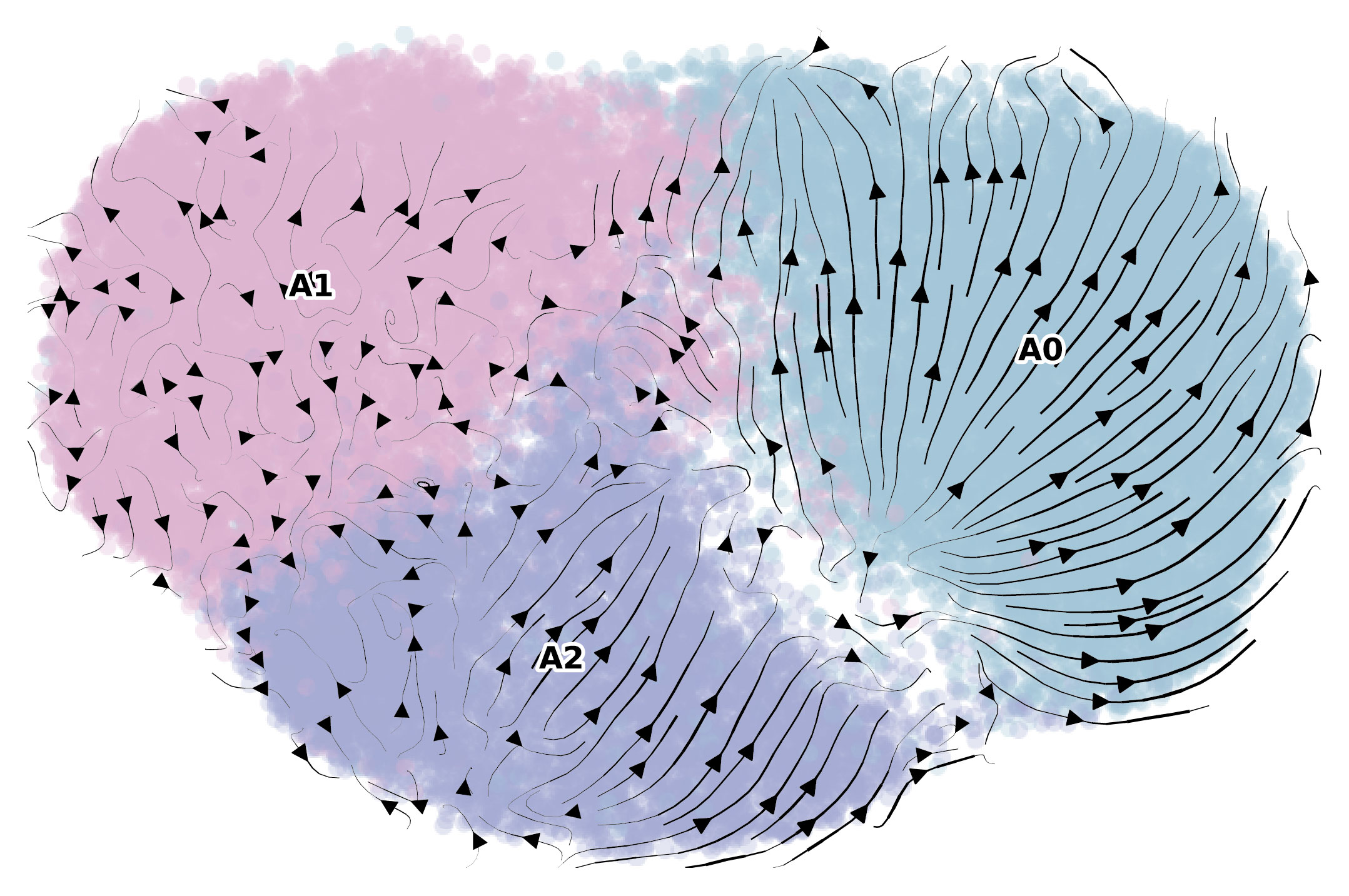


**Supplementary Figure 1.** RNA velocity analysis illustrating the dynamic transitions among murine adipocyte nuclei subclusters.


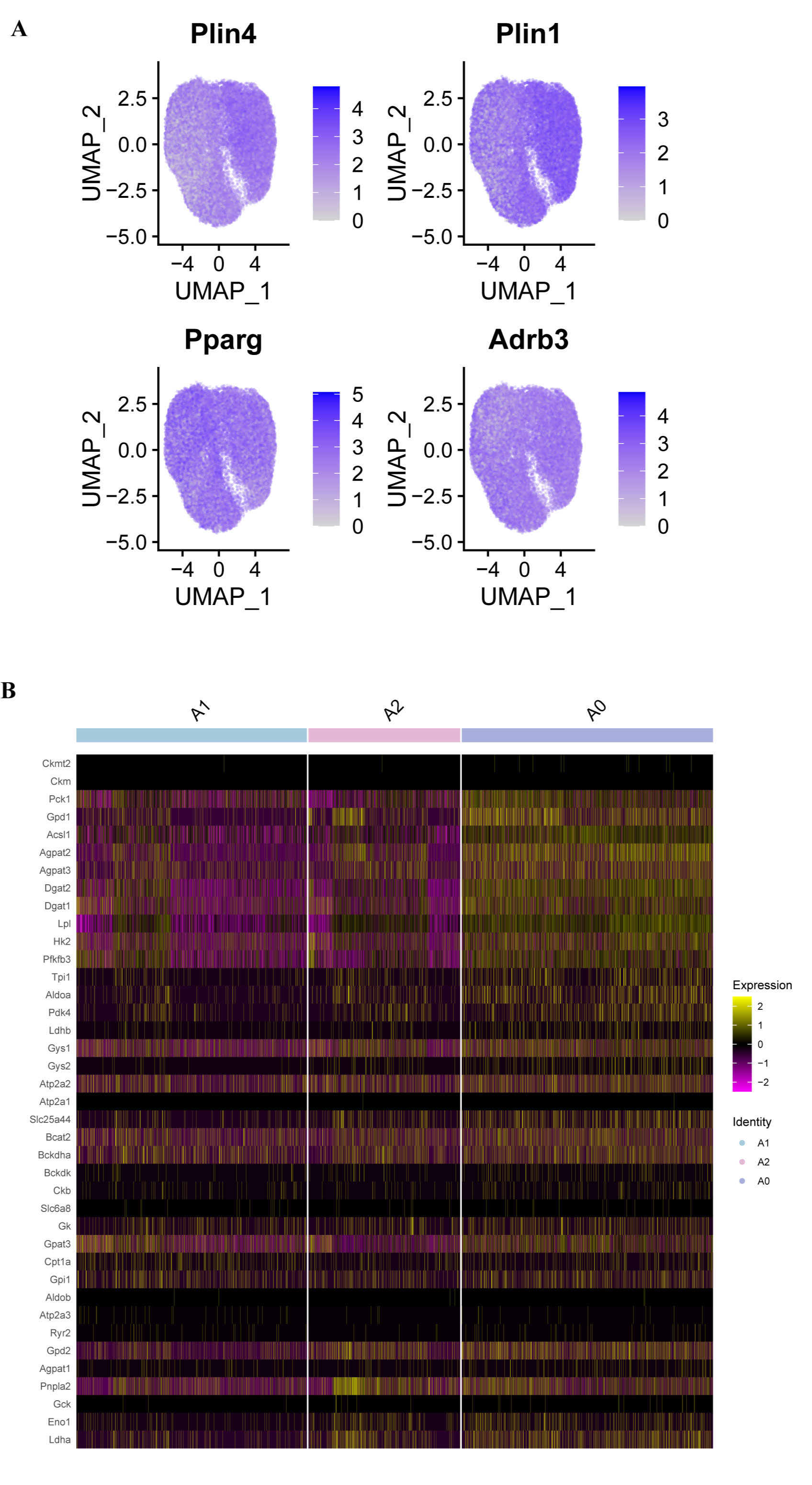


**Supplementary Figure 2.** Characterization of adipocyte subpopulations in mouse eWAT using snRNAseq. **(A)** Feature plots for classic adipocyte markes. **(B)** Heatmap of futile cycle related genes for each population in integrated analysis.


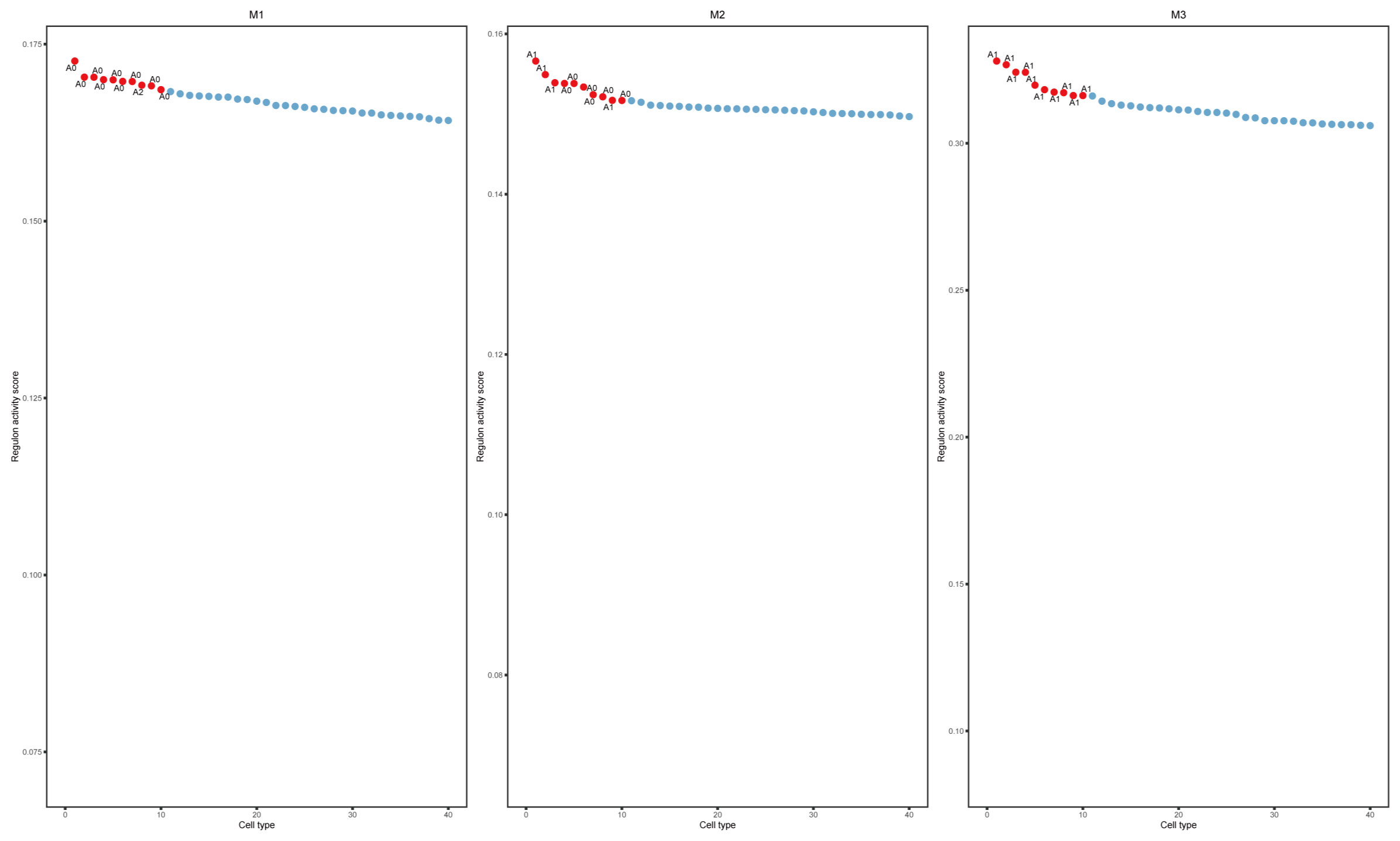


**Supplementary Figure 3.** Scatter plot of module transcriptional regulon activity scores ranked by different cell types.


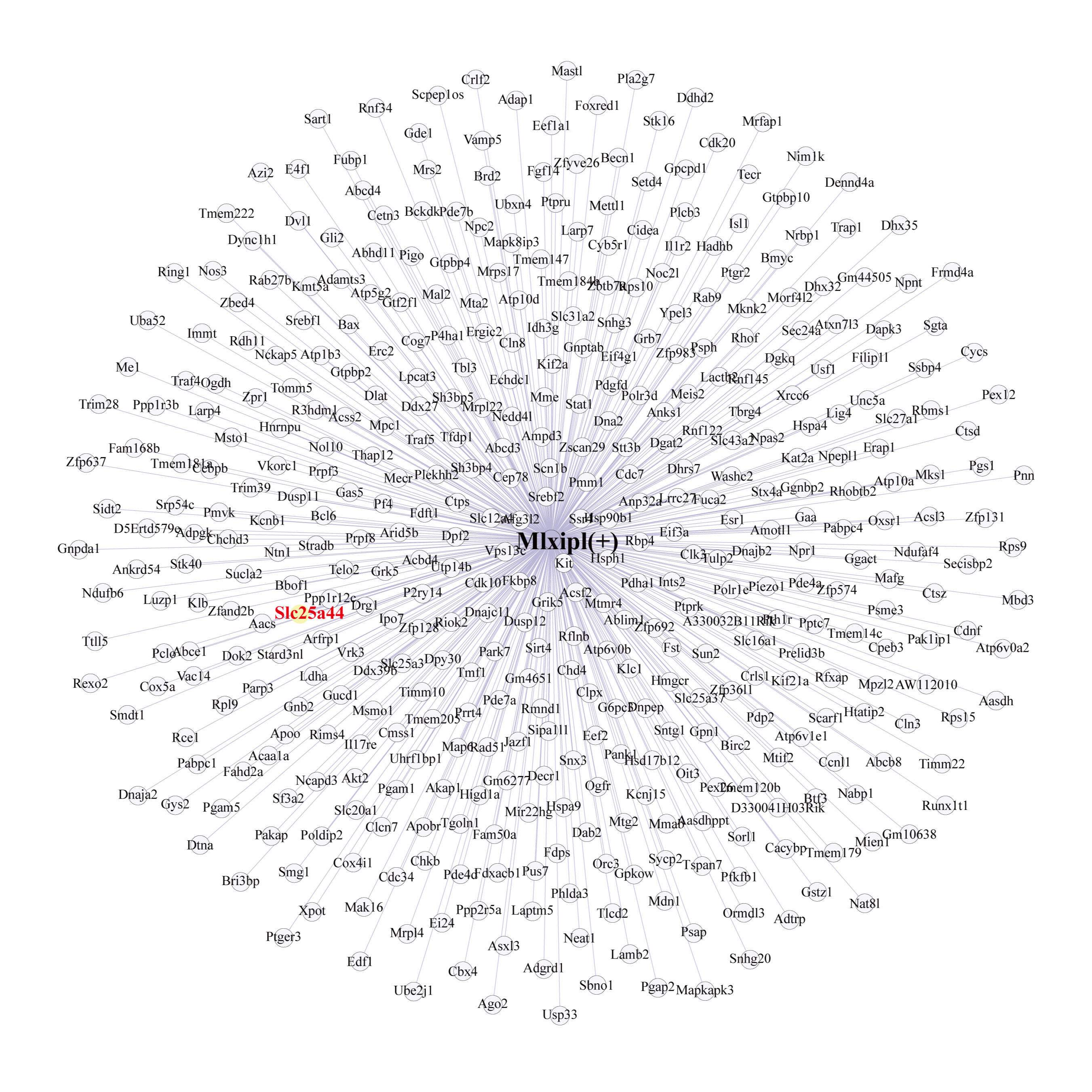


**Supplementary Figure 4.** The regulatory relationship between Mlxipl and its target genes.

## Supplementary Tables

Supplementary Tables can be found in the Supplementary Table S1 and Supplementary Table S2 file.
